# Variation in Colostrum Brix Values and Associations Between Initial Body Weight, Colostrum Intake and Piglet Survival from 24 h to Weaning in a Swedish Sow Herd

**DOI:** 10.3390/ani16213326

**Published:** 2026-10-22

**Authors:** Anna Carlertz, Elin Skans, Rebecka Westin, Magdalena Jacobson

**Affiliations:** 1 Department of Clinical Sciences, Swedish University of Agricultural Sciences, POB 7054, SE-750 07 Uppsala, Sweden; magdalena.jacobson@slu.se; 2 Ambulatory Clinic, SLU University Animal Hospital, Swedish University of Agricultural Sciences, POB 7054, SE-750 07 Uppsala, Sweden; elnskn@hotmail.se; 3 Department of Applied Animal Science and Welfare, Swedish University of Agricultural Sciences, POB 234, SE-532 23 Skara, Sweden; rebecka.westin@slu.se

**Keywords:** colostrum, Brix, sow, piglet, survival

## Abstract

Colostrum is essential for neonatal piglet survival, but individual teat measurements may not represent sow-level mean Brix values. Brix refractometry provides an indirect on-farm estimate of colostrum IgG concentration. This exploratory study quantified Brix variation within and between sows and examined associations between initial body weight, sow Brix values, estimated colostrum intake, and survival from approximately 24 h to weaning. Colostrum was sampled from 28 sows in a Swedish high-health herd. A total of 410 teat samples were analysed and 373 piglets were weighed at colostrum sampling and followed until weaning. The mean sow Brix value was 27.6%. Approximately half the variation was attributable to differences between sows, with the remainder occurring between teats within sows. Empirical resampling analysis showed that sampling multiple teats improved the precision of sow-level mean Brix estimates; with five teats, 95.2% of estimates were within ±1.5 Brix percentage points of the observed sow mean. No clear association between sow-level Brix values and survival from 24 h to weaning was detected. Survival was associated with initial body weight, and the association with estimated colostrum intake depended on initial body weight, being strongest for piglets with lower body weight. Given limited sow numbers and single-herd design, these findings require multi-herd validation.

## Simple Summary

Newborn piglets depend on colostrum intake for energy supply, immune protection, and survival. Brix refractometry can be used on farms to estimate colostrum Ig G concentration, but whether measurements can reliably predict subsequent piglet performance remains insufficiently investigated. In this study, colostrum was sampled from 28 sows, and 373 piglets were weighed within 3 h after birth. Among piglets surviving to 24 h, survival to weaning was mainly related to initial body weight, and higher estimated colostrum intake appeared most important for the smallest piglets. Brix values varied considerably between teats within the same sow, indicating that a single sample may not adequately represent the sow’s overall colostrum mean Brix value. An empirical resampling analysis indicated that sampling multiple teats improved the precision of sow-level mean Brix estimates, although the optimal number requires further validation. No clear association between sow-level Brix values and piglet survival was detected in this herd.

## 1. Introduction

Worldwide, the pre-weaning piglet mortality rate ranges from 11% to 24% [1]; thus, reducing mortality can have a substantial economic and animal welfare impact [2,3]. Among the most well-documented factors associated with piglet survival are birth weight and colostrum intake [4,5,6]. Early colostrum intake is essential for passive immune transfer, energy supply, thermoregulation, and survival in neonatal piglets [7].

Piglets are born without antibodies as immunoglobulins are not transferred across the placenta. Thus, the passive transfer of antibodies through colostrum is necessary to establish protection against environmental pathogens and enhance neonatal piglet health and survival [7,8,9,10]. Colostrum is also important for several other reasons. Newborn piglets lack brown adipose tissue and have very limited energy reserves at birth [7,11]. Colostrum must therefore also provide the essential energy needed to support thermoregulation, physical activity, and early postnatal growth [7].

Colostrum consists of dry matter, proteins, fat, lactose, minerals, and growth factors, as well as antibacterial components such as cytokines, oligosaccharides, lactoferrin, and lactoperoxidase [12,13,14,15,16]. The dry matter and protein contents in colostrum are high, whereas lactose and fat are present at lower levels. The elevated protein content is primarily due to the high concentration of immunoglobulins present in colostrum [12,17].

Colostrum is the first secretion from the mammary gland and is largely produced prior to parturition [18]. It is released during suckling within the first 24 h after birth of the first piglet. Between 12 and 48 h post-partum, colostrum gradually transitions into mature milk [19]. Kielland et al. [20] showed a strong association between colostrum immunoglobulin G (Ig G) and piglet plasma Ig G levels and that plasma concentrations of Ig G in the first hours after birth are positively correlated with survival [21]. Thus, piglet colostrum intake and colostrum Ig G concentration are relevant factors associated with piglet survival.

Litter size has increased in recent years [22], but there is no correlation between litter size and the amount of colostrum produced by the sow. However, there is a negative correlation between the litter size and the amount of colostrum available per piglet [23]. Previous research has shown that colostrum intake is associated with piglet survival [5,6]; however, as litter sizes continue to increase, the availability of colostrum per piglet may become limited [23,24]. Despite the well-recognised importance of colostrum, knowledge is still lacking regarding how individual piglet characteristics, including birth weight, may influence the relationship between colostrum intake and subsequent survival.

Brix refractometry has been suggested as a practical, inexpensive, and rapid tool for estimating the Ig G concentration in sow colostrum at the herd level [25]. Brix refractometry is designed to measure the percentage of sucrose in liquids; when applied to fluids without sucrose, such as colostrum, it provides an approximate estimate of total solids content, expressed as a Brix value (%) [26]. Hasan et al. [25] validated this method by comparing Brix values with ELISA-determined Ig G concentrations, showing a strong correlation when samples were collected within 3 h post-partum. They emphasised that delayed sampling reduced the correlation between Brix values and true Ig G levels, as the immunoglobulin content declines more rapidly than the total solid content that can be detected by the refractometer. Therefore, early sampling is critical for accurate estimation of the colostrum Ig G content. These authors also proposed threshold values for interpreting Brix measurements taken during the first hours of colostrum production. A Brix value below 20% was considered “Poor”, 20–24% “Borderline”, 25–29% “Adequate”, and ≥30% “Very Good” [25].

Birth weight and colostrum intake are well-established factors associated with piglet survival, but their relative importance and potential interaction in relation to subsequent survival warrant further investigation [4,5,6,24]. Although Brix refractometry has been validated as an indirect measure of sow colostrum Ig G concentration, evidence is limited regarding whether sow-level Brix values are associated with piglet survival. Moreover, within-sow variation in Brix values among individual teats has received limited attention, making it uncertain how representative individual colostrum samples are of the sow-level mean. These questions are relevant for both the biological interpretation and practical use of Brix measurements under field conditions.

The primary objectives of this study were: (1) to characterise within- and between-sow variation in colostrum Brix values and assess the representativeness of individual teat samples for the sow-level mean; (2) to evaluate associations between initial body weight, estimated colostrum intake, sow-level mean colostrum Brix values, and piglet survival from approximately 24 h to weaning. We hypothesised that lower initial body weight recorded within three hours after birth, lower estimated colostrum intake, and lower sow-level mean colostrum Brix values would be associated with reduced piglet survival during this period.

## 2. Materials and Methods

The study was approved by the Ethics Committee for Animal Experimentation in Uppsala, Sweden (Dnr. 5.2.18-02900/2020).

### 2.1. Animals and Housing

The study was conducted in six farrowing batches on the pig herd at the Lövsta Agricultural Research Centre, Swedish University of Agricultural Sciences (SLU), Uppsala, Sweden, between August and October 2022 and in July 2023. The herd is a specific pathogen-free (SPF), closed herd of approximately 100 sows, with all replacement gilts bred within the herd. The farm kept detailed records, monitoring animals at the individual level.

Initial body weight was recorded for 373 piglets from 28 litters in which colostrum samples were collected from the sows. Piglets surviving to approximately 24 h were weighed again, and body weight at weaning (5 weeks of age) was recorded for piglets remaining in the study. Sow parity ranged from 1 to 8, and all sows included in the study were Yorkshire × Landrace crossbreds (Topigs Norsvin). The sows were group-housed on deep straw-litter bedding during gestation and housed in free-farrowing pens during farrowing and lactation. During the farrowing and lactation period, the sows were fed a commercial complete diet formulated for lactating sows four times daily. The diet contained 13.2 MJ metabolizable energy/kg and 9.9 MJ net energy (NE)/kg, 157 g crude protein/kg, 9.6 g lysine/kg, 43 g crude fat/kg, 41 g crude fibre/kg, 7 g calcium/kg, 5 g phosphorus/kg, and 2 g sodium/kg. The full lactation allowance corresponded to 21 MJ NE per sow plus 5.6 MJ NE per nursing piglet per day. Individual feed intake was not recorded. Approximately 20 kg of chopped straw was provided as a single allocation approximately 2 days before expected farrowing to provide material for nest-building. Thereafter, straw was replenished as needed to ensure that the sows had continuous access to straw. The farm practiced batchwise farrowing every second week with all-in, all-out in farrowing units of 12 pens per room. Farrowing was not induced, and thus, the sows farrowed spontaneously at any time throughout the 24-h cycle. The onset of farrowing was defined as the birth of the first piglet. Farrowing duration was not systematically recorded.

Cross-fostering was performed after the approximately 24-h weighing. Piglets were transferred only when both the donor and recipient sow were within 48 h after farrowing. Piglets were moved only when a recipient sow with available and functional teats was present, and the piglets moved were those judged able to adapt to a new sow. In total, 16 piglets from six study litters were cross-fostered to other litters and were therefore excluded from all descriptive and statistical analyses, except for the description of initial body weight.

### 2.2. Colostrum Sampling

Colostrum sampling was conducted during predefined 24-h periods when research personnel were available to perform the sampling procedures. During these sampling days, the farrowing unit was monitored at intervals not exceeding 3 h throughout the day and night to enable sampling irrespective of the time of farrowing. When farrowing had started since the preceding inspection, colostrum sampling was performed immediately. If the birth of the first piglet was not directly observed, the exact time of farrowing onset was unknown, but was known to have occurred within the preceding 3 h because farrowing had not started at the previous inspection. All sows that initiated farrowing during a monitored sampling day were eligible for inclusion. In total, 28 sows initiated farrowing during these monitored days, and all 28 were included in the study. Colostrum samples were manually collected from all teats from which colostrum could be obtained (9–16 samples per sow). A predefined inclusion criterion was that colostrum samples had to be collected within 3 h after the onset of farrowing, and all 28 sows fulfilled this criterion. Each colostrum sample was measured immediately using a digital Brix refractometer (PAL-1 BLT/A+W, Atago, Tokyo, Japan). A 0.3 mL sample was placed on the prism and measured on a 0–53% scale with an accuracy of ±0.2%. The prism was wiped clean with a paper towel between each measurement.

### 2.3. Estimation of Colostrum Intake

Piglets were individually marked and first weighed using a digital scale with an accuracy of ±2 g (WE2108, Profilvågen, 2010, Borlänge, Sweden) at the time of colostrum sampling. As sampling was performed within 3 h after the onset of farrowing, the interval between individual birth and first weighing varied. Piglets remained with the sow before sampling and could therefore have suckled before the first weighing. Exact individual times from birth to first weighing and to first suckling were not recorded. The first recorded weight is therefore referred to as the initial body weight rather than pre-colostrum birth weight. Piglets were weighed again approximately 24 h later.

Colostrum intake was calculated using the formula described by Devillers et al. [19]:

$$\begin{array}{cc} Colostrum\;intake & =-217.4+0.217t+\frac{1861019BW}{t}\\ & +\;{BW}_{B}\left(54.80-\frac{1861019}{t}\right)\left(0.9985-3.7\times 10^{-4}t_{FS}+6.1\times 10^{-7}t_{FS}^{2}\right) \end{array}$$

where *BW* is body weight at the second weighing, *BW*_*B*_ is body weight at birth, *t*_*FS*_ is the interval from birth to first suckling (min), and *t* is piglet age at the time of colostrum intake estimation (min). As *t*_*FS*_ was not recorded, it was set to 30 min, consistent with the value used by Devillers et al. [23]. The initial body weight recorded at colostrum sampling was used for *BW_B_*. Exact individual ages at the second weighing were not recorded. As the second weighing was performed approximately 24 h after the initial weighing, which itself occurred within 3 h after birth, t was standardised to 1500 min (25 h), which is within the 17–25 h range for which the equation was developed [19]. These assumptions should be considered when interpreting the estimated colostrum intake.

### 2.4. Piglet Survival

Survival to weaning was recorded for all piglets. Piglets were weighed at approximately 5 weeks of age (at weaning) for descriptive purposes. The mean weaning age was 32 days.

### 2.5. Statistical Analysis

Data were processed and analysed using Stata/IC 14.2 (StataCorp LLC, College Station, TX, USA). Descriptive statistics were used to summarise sow-, litter-, and piglet-level characteristics. The hierarchical structure of the data was considered in all statistical analyses. Individual teat samples represented repeated measurements within sows, whereas piglets represented individual observations nested within litters. For sow-level mean colostrum Brix values, the independent exposure units were the 28 sows, as all piglets within a litter shared the same sow-level Brix value.

For colostrum Brix measurements, descriptive analyses included the distribution of individual teat samples and sow-level summary measures (mean, median, standard deviation [SD], minimum, and maximum). Graphical exploration was performed using boxplots and scatterplots to assess variation between sows, between teat positions, and across parity levels. The proportion of the total variation in individual teat Brix values attributable to differences between sows was estimated using an empty random-intercept mixed-effects model with individual teat Brix as the outcome variable and Sow ID as a random intercept, applying the MIXED command in Stata. From this model, the intraclass correlation coefficient (ICC) was calculated using the estat icc command in Stata.

Differences in colostrum Brix values between teat-pair positions were investigated using a linear mixed-effects model, with teat-pair position included as a categorical fixed effect (eight levels, corresponding to pairs of teats at the same position on the left and right sides of the udder, numbered from anterior to posterior) and sow as a random intercept. The overall association was assessed using a Wald test.

To evaluate how the number of sampled teats affected the precision of sow-level mean Brix estimates, an empirical resampling analysis was performed. For each sow, 1–8 teat samples were randomly selected without replacement, with 1000 repetitions for each sample size. The mean Brix value of each randomly selected sample was compared with the mean calculated from all available teat samples from the same sow. Precision was assessed using the mean absolute error and the proportion of estimates falling within ±1.5 Brix percentage points of the observed sow-level mean. The ±1.5 Brix percentage-point tolerance was selected as an exploratory precision criterion and does not represent a biologically validated threshold.

Associations between estimated colostrum intake and survival to weaning among piglets surviving to the 24-h weighing were analysed using mixed-effects logistic regression. Piglet was the observational unit, and Litter ID was included as a random intercept to account for clustering of piglets within litters. Analyses were performed using the MELOGIT command in Stata.

Following initial exploratory model building, a parsimonious multivariable model was used to reduce the risk of overfitting, given the limited number of deaths. The model included three variables of primary interest: initial body weight (per 100 g increase; centred around the overall mean), estimated colostrum intake (g; centred around the overall mean), and mean sow colostrum Brix value (%; centred around the overall mean). An interaction term between initial body weight and estimated colostrum intake was included to investigate whether the association between colostrum intake and survival differed according to initial body weight.

The interaction was interpreted using model-based predicted probabilities of survival at selected values of initial body weight and estimated colostrum intake. Predictions were calculated from the fixed-effects component of the model, at the mean sow colostrum Brix value and with the random intercept set to zero. The corresponding 95% confidence intervals were calculated on the logit scale and back-transformed to probabilities.

To evaluate the robustness of the interaction between initial body weight and estimated colostrum intake, sensitivity analyses were performed by additionally adjusting for the number of piglets born alive or sow parity, and by including cross-fostered piglets.

Piglets that died before 24 h of age (*n* = 14) were excluded from the primary analysis because colostrum intake could not be estimated for these animals. Cross-fostered piglets (*n* = 16) were also excluded from the primary survival analysis because they were subsequently reared under a different nursing sow and litter environment. Their inclusion was evaluated in a sensitivity analysis. The final analytical dataset regarding piglet survival thus comprised 343 piglets from 28 litters, of which 37 died between 24 h of age and weaning.

## 3. Results

### 3.1. Study Population

A total of 373 piglets were weighed at colostrum sampling. Sixteen piglets were cross-fostered to another litter and were excluded from analyses of survival to weaning, leaving 357 piglets. Of these, 14 died before the second weighing at approximately 24 h of age and therefore had no estimated colostrum intake. Consequently, 343 piglets from 28 litters were included in analyses of estimated colostrum intake and survival from approximately 24 h to weaning. Of these, a further 37 piglets died before weaning, leaving 306 piglets with body weight recorded at weaning (approximately 5 weeks of age).

The mean (±SD) initial body weight was 1.42 ± 0.34 kg, which increased to 1.49 ± 0.37 kg after 24 h and 10.93 ± 2.51 kg at weaning (Table 1). The mean calculated colostrum intake was 275 ± 120 g.

### 3.2. Variation in Colostrum Brix Values

A total of 410 individual colostrum samples collected from 28 sows were available for Brix analysis (Table 2). The mean colostrum total solid content (Brix values) per sow ranged from 24.1 to 32.3%, with an overall mean of 27.6%. None of the sows had a mean colostrum Brix value below 20% and only three sows had mean Brix values below 25%, whereas four sows had values ≥ 30%. Consequently, the interpretative thresholds proposed by Hasan et al. [25] resulted in very small comparison groups in the present dataset.

**Figure 1:**
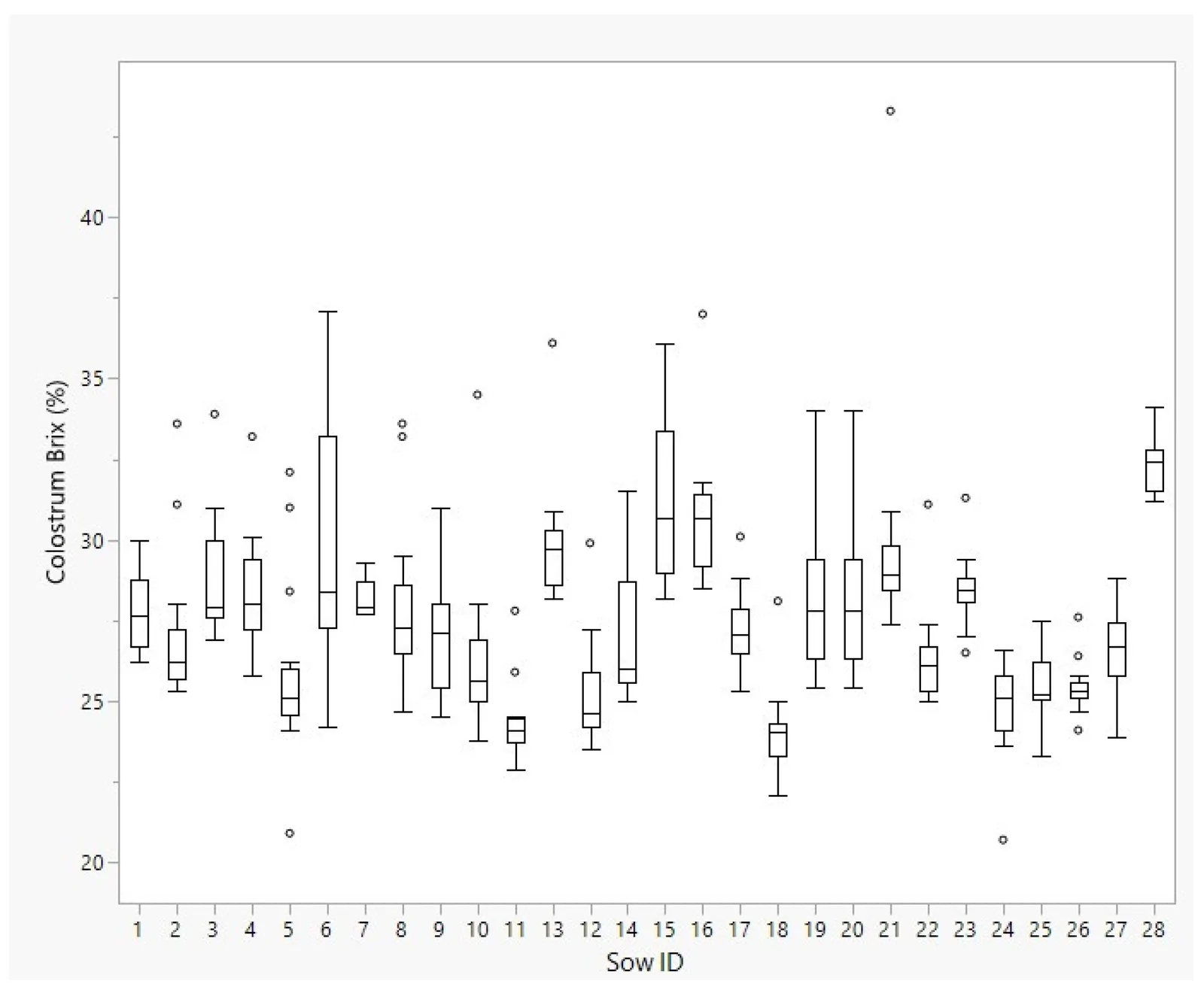
Distribution of individual colostral total solid content (Brix values) by sow (Sow ID). A total of 410 teat samples (9–16 samples per sow) were collected within 3 h after the birth of the first piglet from 28 sows. Boxplots show the median, interquartile range, most extreme observations within 1.5 × IQR (whiskers), and outliers (ο). Considerable variation in colostral Brix values was observed within and between sows.

**Figure 2:**
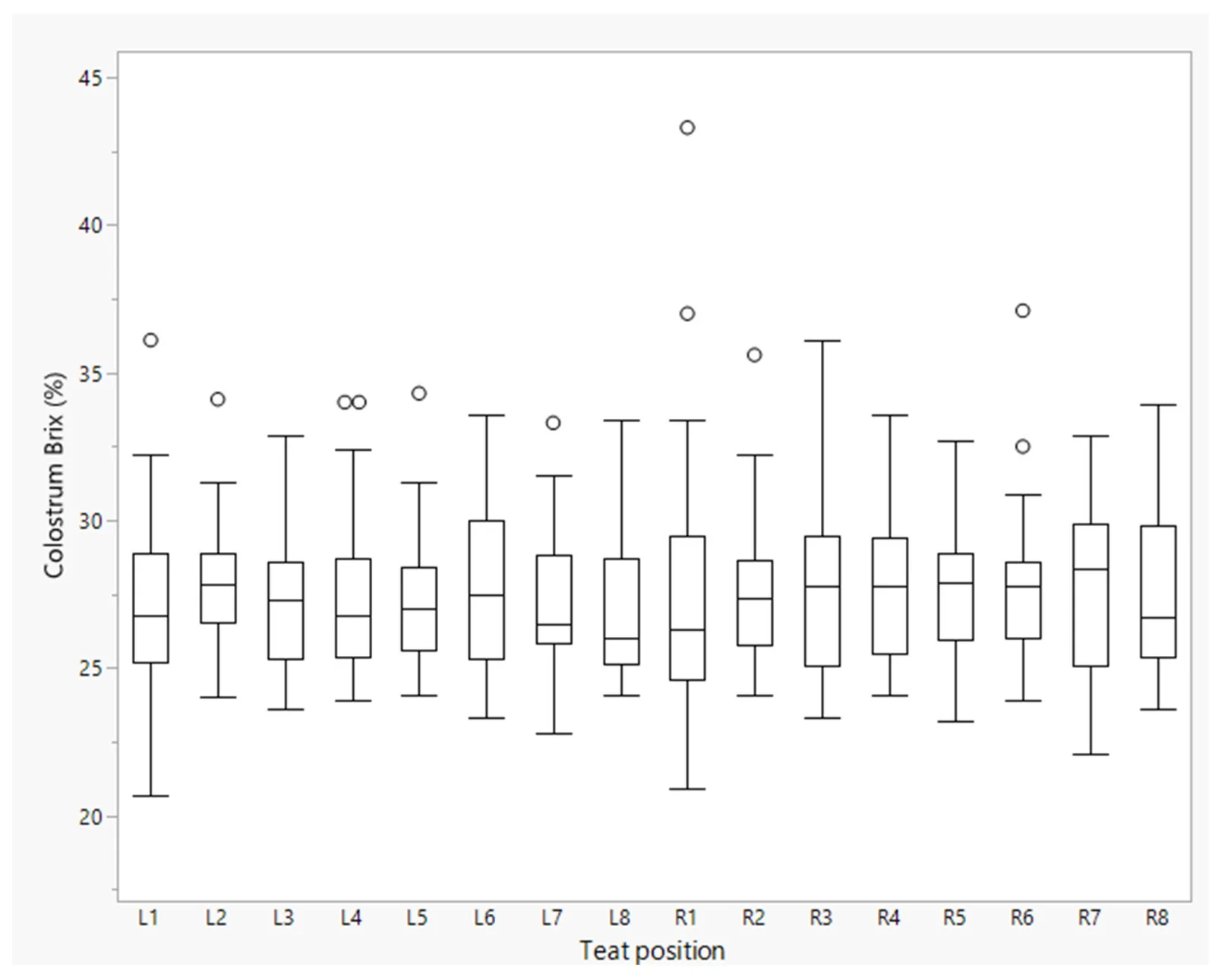
Distribution of individual colostral total solid content (Brix values) by teat position on the left (L1–L8) and right (R1–R8) sides of the udder in 28 sows (410 teat samples; 9–16 teat samples per sow). Colostrum was sampled within 3 h after the birth of the first piglet. Boxplots show the median, interquartile range, 1.5 × IQR (whiskers), and outliers (ο). No apparent differences in colostral Brix values were observed between teat positions.

**Figure 3:**
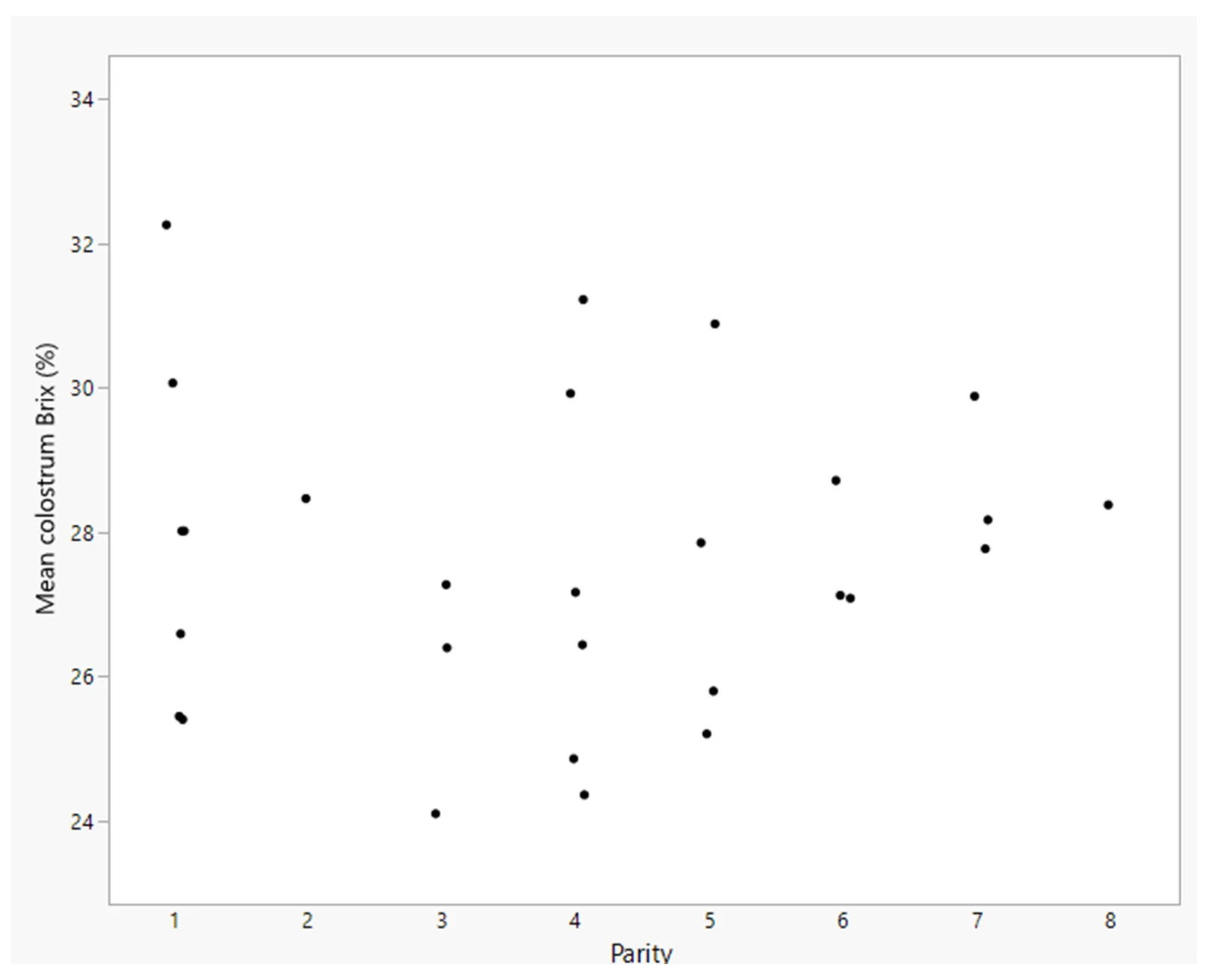
Mean colostral total solid content (Brix values) by parity in 28 sows. Each dot represents the mean Brix value calculated from 9 to 16 teat samples collected within 3 h after the birth of the first piglet. No apparent trend in mean colostral Brix values was observed across parity levels.

Analysis of the individual teat samples demonstrated substantial within-sow variation in colostrum Brix values (Figure 1). Approximately half of the total variability was attributable to differences between sows (ICC = 0.49, 95% CI: 0.35–0.64), whereas the remaining variability occurred between teats within the same sow. Visual inspection of the data revealed no apparent trends in colostrum Brix values according to teat position (Figure 2) which was supported by the absence of a statistically significant association between teat-pair position and Brix value (χ^2^(7) = 2.07, p = 0.956). Similarly, no apparent trend was observed in relation to sow parity (Figure 3).

The empirical resampling analysis showed that the precision of estimated sow-level mean Brix values improved with increasing numbers of sampled teats. When four, five, or six teats were randomly selected, 92.3%, 95.2%, and 97.7% of the estimated means, respectively, were within ±1.5 Brix percentage points of the mean calculated from all available teat samples from the same sow. The corresponding mean absolute errors were 0.66, 0.56, and 0.49 Brix percentage points, respectively.

### 3.3. Associations Between Estimated Colostrum Intake and Piglet Survival from 24 h to Weaning

Among the 357 non-cross-fostered piglets, 14 (3.92%) died before the approximately 24 h weighing (Table 1). Of the 343 piglets surviving to this weighing, 37 (10.79%) died before weaning. Overall pre-weaning mortality was 14.29% (51/357). The survival analysis was restricted to the 343 piglets surviving to the 24-h weighing, for which estimated colostrum intake was available. These piglets originated from 28 litters, and the final mixed-effects logistic regression model included four fixed-effect coefficients for the explanatory variables, corresponding to approximately 9.3 deaths per coefficient (37/4), excluding the intercept and the random-effect variance parameter.

In the mixed-effects logistic regression model, initial body weight was positively associated with survival from 24 h to weaning at the mean estimated colostrum intake (OR per 100 g: 1.39, 95% CI: 1.17–1.67; *p* < 0.001). A significant interaction between estimated colostrum intake and initial body weight was observed (*p* = 0.032), indicating that the association between colostrum intake and survival depended on initial body weight. Mean sow colostrum Brix value was not clearly associated with survival (OR per percentage point: 0.92, 95% CI: 0.71–1.18; *p* = 0.509).

**Figure 4:**
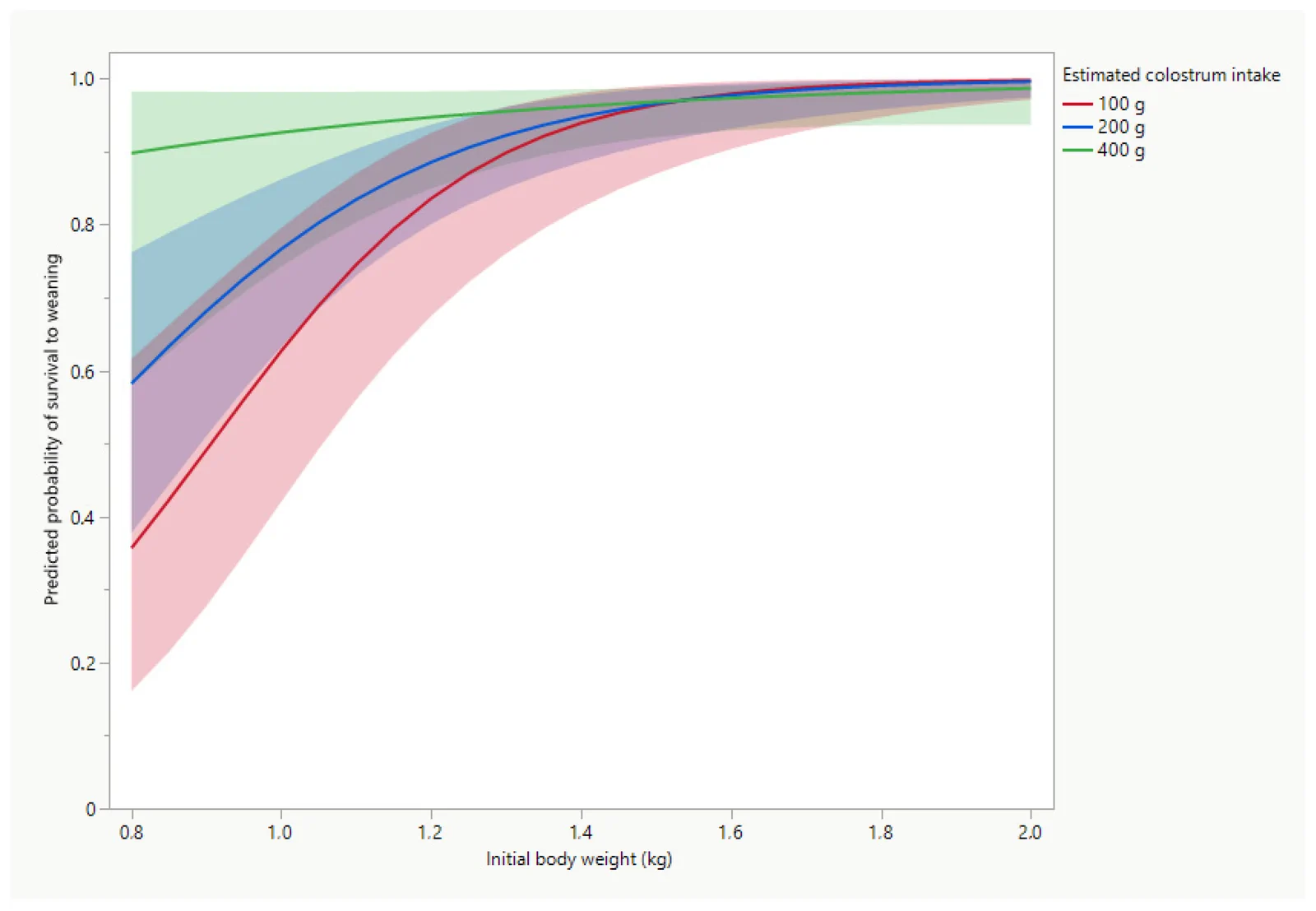
Predicted probability of survival from 24 h after birth to weaning (*n* = 343 piglets from 28 litters) in relation to initial body weight at three levels of estimated colostrum intake. Predictions were obtained from a mixed-effects logistic regression model including initial body weight, estimated colostrum intake and their interaction, and sow colostrum Brix value, with litter included as a random intercept. Predictions were calculated at the mean sow colostrum Brix value. Lines represent estimated colostrum intakes of 100, 200, and 400 g, while shaded areas represent 95% confidence intervals.

The predicted probabilities from the model are shown in Figure 4. For piglets with an initial body weight of 0.8 kg, the predicted probability of survival increased from 36% (95% CI: 16–62%) at an estimated colostrum intake of 100 g to 58% (95% CI: 38–76%) at 200 g. At an initial body weight of 1.6 kg, the corresponding predicted probabilities were 98% (95% CI: 90–100%) and 98% (95% CI: 93–99%), respectively.

In sensitivity analyses, the interaction between initial body weight and estimated colostrum intake remained negative when adjusting for number of piglets born alive or sow parity, and when cross-fostered piglets were included. The interaction remained statistically significant in most analyses, although it was slightly above the significance threshold when adjusting for sow parity (*p* = 0.052). The interaction also remained similar when cross-fostered piglets were included and cross-fostering status was added to the model (OR: 0.99879, 95% CI: 0.99771–0.99988; *p* = 0.029).

The 14 piglets that died before 24 h of age had a mean initial body weight of 1.00 ± 0.40 kg (median 0.91 kg, range 0.50–1.61 kg) compared with 1.44 kg (median 1.46 kg, range 0.63–2.28) among piglets included in the survival analysis.

## 4. Discussion

Brix refractometry has previously been validated as a practical on-farm method providing an indirect estimate of colostrum Ig G concentration in sows [25]. Colostrum Ig G was not measured directly in the present study; the method has, however, also been validated against Ig G concentration in cows (r = 0.83), goats (r = 0.83) and sheep (r = 0.75) [27], and Brix values were therefore considered a proxy for colostrum Ig G concentration. Brix refractometry provides an indirect measure of total solids and should not be equated with overall colostrum quality. However, accurate interpretation requires that colostrum samples are collected during the first three hours after the onset of farrowing to obtain a strong association between Brix values and colostrum Ig G concentration [25]. The present study shows a considerable variation in Brix values both between sows and between teats within the same sow. Approximately half of the total variation was attributable to differences between sows, while the remaining variation occurred between teats within sows.

The ICC of 0.49 indicates that a Brix measurement from an individual teat sample may provide an imprecise estimate of the sow-level mean. Given the limited number of sows, the ICC estimate should be interpreted with caution and requires confirmation in larger multi-herd studies. No statistically significant association was found between teat-pair position and Brix values, suggesting that sampling from specific teat positions may not be necessary. Nevertheless, samples from several teats are preferable when the aim is to estimate the mean Brix value of a sow.

Because samples were collected up to 3 h after the onset of farrowing, some teats may have been partially emptied by suckling piglets before sampling. Consequently, the measured Brix value may not fully reflect the colostrum present in the mammary gland at the onset of farrowing. Prior suckling could therefore have contributed to the observed teat-to-teat variation and represents a potential source of measurement error. The magnitude and direction of this effect could not be determined in the present study. However, prior suckling before sampling is also likely to occur under field conditions and should therefore be considered when interpreting Brix measurements obtained in practice. Thus, both the timing of sampling and the number of teats sampled need to be considered when Brix refractometry is used as an indirect estimate of sow colostrum Ig G concentration. The empirical resampling analysis indicated that sampling approximately 4–6 teats could improve the precision of sow-level mean Brix estimates, with 92.3–97.7% of estimates falling within ±1.5 Brix percentage points of the observed sow-level mean. The ±1.5 Brix percentage-point tolerance used in this analysis was an exploratory precision criterion rather than a biologically validated threshold. However, variation in sampling time and potential differences in prior suckling between teats may have influenced the observed within-sow variation. Furthermore, the analysis did not evaluate pooling of samples. The optimal number of teats to sample therefore remains uncertain, and the findings should not be interpreted as a validated sampling recommendation. Confirmation in larger multi-herd studies is required.

The importance of adequate colostrum intake for neonatal piglet survival and health has been demonstrated in numerous studies [7,24,28]. Previous studies have shown that colostrum Ig G concentration is closely related to piglet plasma Ig G concentration, and that plasma Ig G concentrations during the first hours after birth are positively associated with piglet survival [20,21]. In the present study, no clear association was found between sow-level mean colostrum Brix value and piglet survival from approximately 24 h to weaning among piglets for which colostrum intake could be estimated. Higher initial body weight was associated with survival, and the association between estimated colostrum intake and survival depended on initial body weight. Model predictions indicated that the positive association between estimated colostrum intake and survival was strongest among piglets with lower initial body weight and weaker among heavier piglets. This is consistent with previous studies identifying birth weight and colostrum intake as important correlates of neonatal viability and pre-weaning survival [4,6,7,28]. However, estimated colostrum intake may also reflect overall neonatal vitality, including the ability to reach the udder and suckle, competition for colostrum, and early growth [6,24,29]. Low-vitality piglets may also be more vulnerable to crushing [3,30]. The observed association should therefore not be interpreted as evidence of a causal effect of colostrum intake on survival.

An important limitation is that colostrum intake could only be estimated for piglets surviving until the second weighing at approximately 24 h. Consequently, the 14 piglets that died before this weighing were not included in analyses involving estimated colostrum intake. The survival analysis therefore does not assess total pre-weaning mortality, but survival from the second weighing to weaning among piglets that had survived to the second weighing. This should be considered when interpreting the association between estimated colostrum intake and survival.

A further limitation concerns the initial body weight and standardised time for second weighing used in the estimation of colostrum intake. Because piglets were first weighed at the time of colostrum sampling, some piglets may already have suckled before this measurement. The initial body weight may therefore have exceeded the true pre-colostrum birth weight. Its use as *BW*_*B*_ in the Devillers equation may have resulted in underestimation of estimated colostrum intake. Although 25 h was considered the most appropriate standardised value based on the study protocol, individual variation in age at the second weighing may also have affected the estimated colostrum intake. Exact individual intervals between birth, first suckling, and first and second weighing were not available, and retrospective adjustment was therefore not possible.

Only three sows had Brix values below 25% and four sows had values ≥ 30%, with most sows having values within the 25–29% range. This limited variation may partly reflect the fact that all sows were from the same herd and were kept under similar housing, feeding, and management conditions [31,32]. Hasan et al. also suggested that herd-related differences could contribute to variation in colostrum composition between herds [25]. Although individual piglets were included as observations in the mixed-effects model, the independent exposure units for sow-level mean colostrum Brix values were the 28 sows. The limited number of independent exposure units and the relatively narrow between-sow range in Brix values limited the precision of the estimated association with survival. Thus, the present data do not exclude a biologically relevant association between sow-level Brix values and piglet survival. The present study could not evaluate between-herd variation but provides information on Brix variation within a single high-health herd. Furthermore, genetic background has been reported to influence colostrum composition [33] but all the sows in the present study were Yorkshire × Landrace crossbreds. Therefore, genotype-related effects on colostrum composition could not be evaluated.

Increasing litter sizes remain an important challenge in current production systems. Larger litters may reduce individual birth weights, prolong farrowing, and increase competition at the udder, increasing the likelihood that later-born piglets access less colostrum and have lower Ig G concentrations [29]. The observed interaction suggests that the association between estimated colostrum intake and survival may be stronger among piglets with lower initial body weight. However, the relatively small number of litters (*n* = 28) and deaths between 24 h and weaning (*n* = 37) may have limited the precision and stability of the estimated associations, particularly the interaction effect. This finding requires confirmation in larger studies before specific management recommendations based on birth weight can be made.

A strength of the present study was the intensive sampling of 9–16 teats per sow within three hours after the onset of farrowing, which made it possible to quantify variation within and between sows. Piglets were also followed from birth until weaning. However, the relatively small number of sows (*n* = 28) and the single-herd design limit the precision and generalisability of the sow-level estimates. Although 410 individual teat samples were analysed, these represented repeated measurements nested within 28 sows and should not be considered independent biological observations. Data collection was conducted during two defined sampling periods (August–October 2022 and July 2023), and the study was not designed to evaluate seasonal variation. Therefore, potential seasonal effects on colostrum Brix values and litter characteristics cannot be excluded. Accordingly, the statistical associations should be interpreted as exploratory estimates within this study population rather than as population-level effects.

Within the study herd, on-farm measurement of sow colostrum Brix values provided information on within- and between-sow variation. Brix refractometry provides a simple, objective, and practical on-farm method for indirectly estimating colostrum Ig G concentration [25]. In addition to offering rapid results, it enables the evaluation of whether management interventions have a measurable effect on colostrum composition. However, the present findings demonstrate that the practical value of these measurements depends not only on sampling time but also on whether the samples adequately represent the udder. In herds where farrowing is not induced, obtaining samples within the optimal time frame requires continuous surveillance and is labour-intensive. Thus, implementation of Brix testing under field conditions requires consideration of staffing and the feasibility of obtaining samples within the recommended time window. Further studies are needed to validate the number of teat samples required to obtain a reliable sow-level estimate and to determine whether sow-level Brix values are associated with piglet survival across different herds.

## 5. Conclusions

In this single high-health herd, piglet survival from approximately 24 h to weaning was associated with initial body weight, while the association between estimated colostrum intake and survival during this period depended on initial body weight and was most pronounced among piglets with lower weight. No clear association between mean sow colostrum Brix values and survival during this period was detected.

Considerable variation in Brix values was observed among teats within individual sows, indicating that a single-teat sample may not adequately represent the sow-level mean Brix value. The empirical resampling analysis indicated that sampling multiple teats improved the precision of sow-level mean Brix estimates. However, the optimal number of teats remains to be established in larger multi-herd studies.

## Figures and Tables

**Table 1:** Characteristics of the sows and piglets included in the study, including mortality among live-born piglets during different periods.

Variable	*n*	Mean ± SD	Median	Range
Sows and litters				
Parity	28	3.9	4	1–8
Live-born piglets/litter	28	16.9 ± 4.6	16	7–24
Nursing litter size (after cross-fostering)	28	16.7 ± 3.4	16.5	9–24
Weaned piglets/litter	28	13.1 ± 1.8	13	8–19
Piglets				
Initial body weight (kg)	373	1.42 ± 0.34	1.45	0.50–2.28
Colostrum intake (g)	343 ^1^	275 ± 120	259	0–873
Body weight at 24 h (kg)	343 ^1^	1.49 ± 0.37	1.51	0.62–2.47
Body weight at weaning (kg)	306 ^2^	10.93 ± 2.51	11.05	3.3–17.1
Live-born piglet mortality from birth to 24 h (%)	357 ^3^	3.92		
Piglet mortality from 24 h to weaning (%)	343 ^1^	10.79		
Overall pre-weaning mortality (%)	357 ^3^	14.29	–	–

^1^ Difference in *n* reflects mortality before 24 h of age when piglets were weighed to calculate colostrum intake (14 piglets) and cross-fostered piglets removed from the litters (16 piglets). ^2^ Difference in *n* reflects mortality from birth until weaning (51 piglets) and cross-fostered piglets removed from the litters (16 piglets). ^3^ Difference in *n* reflects cross-fostered piglets removed from the litters (16 piglets). –, not applicable.

**Table 2:** Descriptive statistics of separate and sow-level colostrum measurements of the total solid content (Brix value) obtained from 410 teat samples collected from 28 sows within 3 h after the onset of farrowing.

Variable	*n*	Mean ± SD	Median	Range
Single teat measurements (Brix value; %)	410	27.5 ± 2.8	27.25	20.7–43.3
No. of teats sampled per sow	28	14.6 ± 2.0	15	9–16
Mean Brix value per sow (%)	28	27.6 ± 2.1	27.5	24.1–32.3
Within-sow SD (%)	28	1.85 ± 0.83	1.81	0.53–4.08

## Data Availability

The datasets used and analysed during the current study are available from the corresponding author upon reasonable request.

## Abbreviations

The following abbreviations are used in this manuscript:

BW	Body weight
ICC	Intraclass correlation coefficient
Ig G	Immunoglobulin G
OR	Odds ratio
SD	Standard deviation
SPF	Specific pathogen-free

## References

[1] Oliviero C., Rodriguez-Martinez H., Soede N.M., Flowers W.L. (2013). Management to improve neonate piglet survival. Control of Pig Reproduction IX.

[2] Stygar A.H., Chantziaras I., Maes D., Aarestrup Moustsen V., De Meyer D., Quesnel H., Kyriazakis I., Niemi J.K. (2022). Economic feasibility of interventions targeted at decreasing piglet perinatal and pre-weaning mortality across European countries. Porc. Health Manag..

[3] Kielland C., Wisløff H., Valheim M., Fauske A.K., Reksen O., Framstad T. (2018). Preweaning mortality in piglets in loose-housed herds: Etiology and prevalence. Animal.

[4] Jourquin J., Morales J., Bokenkroger C.D. (2016). Impact of piglet birth weight increase on survivability and days to market, a simulation model. J. Anim. Sci..

[5] Devillers N., Le Dividich J., Prunier A. (2011). Influence of colostrum intake on piglet survival and immunity. Animal.

[6] Decaluwé R., Maes D., Wuyts B., Cools A., Piepers S., Janssens G.P.J. (2014). Piglets’ colostrum intake associates with daily weight gain and survival until weaning. Livest. Sci..

[7] Le Dividich J., Rooke J.A., Herpin P. (2005). Nutritional and immunological importance of colostrum for the new-born pig. J. Agric. Sci..

[8] Milon A., Aumaitre A., Le Dividich J., Franz J., Metzger J.J. (1983). Influence of birth prematurity on colostrum composition and subsequent immunity of piglets. Ann. Rech. Vét..

[9] Rooke J.A., Bland I.M. (2002). The acquisition of passive immunity in the new-born piglet. Livest. Prod. Sci..

[10] Bandrick M., Ariza-Nieto C., Baidoo S.K., Molitor T.W. (2014). Colostral antibody-mediated and cell-mediated immunity contributes to innate and antigen-specific immunity in piglets. Dev. Comp. Immunol..

[11] Herpin P., Damon M., Le Dividich J. (2002). Development of thermoregulation and neonatal survival in pigs. Livest. Prod. Sci..

[12] Klobasa F., Werhahn E., Butler J.E. (1987). Composition of sow milk during lactation. J. Anim. Sci..

[13] Albera E., Kankofer M. (2009). Antioxidants in colostrum and milk of sows and cows. Reprod. Domest. Anim..

[14] Tan L., Wei T., Yuan A., He J., Liu J., Xu D., Yang Q. (2017). Dietary supplementation of Astragalus polysaccharides enhanced immune components and growth factors EGF and IGF-1 in sow colostrum. J. Immunol. Res..

[15] Jahan M., Francis N., Wang B. (2020). Milk lactoferrin concentration of primiparous and multiparous sows during lactation. J. Dairy Sci..

[16] Nuntapaitoon M., Juthamanee P., Theil P.K., Tummaruk P. (2020). Impact of sow parity on yield and composition of colostrum and milk in Danish Landrace × Yorkshire crossbred sows. Prev. Vet. Med..

[17] Theil P.K., Lauridsen C., Quesnel H. (2014). Neonatal piglet survival: Impact of sow nutrition around parturition on fetal glycogen deposition and production and composition of colostrum and transient milk. Animal.

[18] Quesnel H., Farmer C., Theil P.K., Farmer C. (2015). Colostrum and milk production. The Gestating and Lactating Sow.

[19] Devillers N., van Milgen J., Prunier A., Le Dividich J. (2004). Estimation of colostrum intake in the neonatal pig. Anim. Sci..

[20] Kielland C., Rootwelt V., Reksen O., Framstad T. (2015). The association between immunoglobulin G in sow colostrum and piglet plasma. J. Anim. Sci..

[21] Vallet J.L., Miles J.R., Rempel L.A. (2013). A simple novel measure of passive transfer of maternal immunoglobulin is predictive of preweaning mortality in piglets. Vet. J..

[22] Prunier A., Heinonen M., Quesnel H. (2010). High physiological demands in intensively raised pigs: Impact on health and welfare. Animal.

[23] Devillers N., Farmer C., Le Dividich J., Prunier A. (2007). Variability of colostrum yield and colostrum intake in pigs. Animal.

[24] Quesnel H., Farmer C., Devillers N. (2012). Colostrum intake: Influence on piglet performance and factors of variation. Livest. Sci..

[25] Hasan S., Junnikkala S., Valros A., Peltoniemi O., Oliviero C. (2016). Validation of Brix refractometer to estimate colostrum immunoglobulin G content and composition in the sow. Animal.

[26] Quigley J.D., Lago A., Chapman C., Erickson P., Polo J. (2013). Evaluation of the Brix refractometer to estimate immunoglobulin G concentration in bovine colostrum. J. Dairy Sci..

[27] Kessler E.C., Bruckmaier R.M., Gross J.J. (2021). Short communication: Comparative estimation of colostrum quality by Brix refractometry in bovine, caprine, and ovine colostrum. J. Dairy Sci..

[28] Le Dividich J., Charneca R., Thomas F. (2017). Relationship between birth order, birth weight, colostrum intake, acquisition of passive immunity and pre-weaning mortality of piglets. Span. J. Agric. Res..

[29] Oliviero C., Junnikkala S., Peltoniemi O. (2019). The challenge of large litters on the immune system of the sow and the piglets. Reprod. Domest. Anim..

[30] Rangstrup-Christensen L., Schild S.-L.A., Pedersen L.J., Sørensen J.T. (2018). Causes of preweaning mortality in organic outdoor sow herds. Res. Vet. Sci..

[31] Decaluwé R., Maes D., Declerck I., Cools A., Wuyts B., De Smet S., Janssens G.P.J. (2013). Changes in back fat thickness during late gestation predict colostrum yield in sows. Animal.

[32] Nuntapaitoon M., Suwimonteerabutr J., Am-in N., Tienthai P., Chuesiri P., Kedkovid R., Tummaruk P. (2019). Impact of parity and housing conditions on concentration of immunoglobulin G in sow colostrum. Trop. Anim. Health Prod..

[33] Farmer C., Charagu P., Palin M.F. (2007). Influence of genotype on metabolic variables, colostrum and milk composition of primiparous sows. Can. J. Anim. Sci..

